# Applying chlorogenic acid in an alginate scaffold of chondrocytes can improve the repair of damaged articular cartilage

**DOI:** 10.1371/journal.pone.0195326

**Published:** 2018-04-05

**Authors:** Xin Cheng, Ke Li, Shengsong Xu, Peizhi Li, Yu Yan, Guang Wang, Zachary Berman, Rui Guo, Jianxin Liang, Sira Traore, Xuesong Yang

**Affiliations:** 1 Department of Histology and Embryology, Joint Laboratory for Embryonic Development & Prenatal Medicine, Medical College, Jinan University, Guangzhou, Guangdong, China; 2 Department of Radiology, University of California San Diego, San Diego, California, United States of America; 3 Key Laboratory for Regenerative Medicine of the Ministry of Education, Jinan University, Guangzhou, Guangdong, China; University of Umeå, SWEDEN

## Abstract

Damaged cartilage has very low regenerative potential which has led to the search for novel tissue-engineering approaches to help treat cartilage defects. While various approaches have been reported, there is no perfect treatment currently. In this study we evaluated the effects of a plant extract, chlorogenic acid (CGA), as part of chondrocyte transplantation on a model of knee joint injury in chicks. First, primary cultured chondrocytes used to evaluate the effects of CGA on chondrogenesis. Then using an articular cartilage injury model of chick knee we assessed the functional recovery after transplantation of the complexes containing chondrocytes and CGA in an alginate scaffold. Histological analysis, PCR, and western blot were further used to understand the underlying mechanisms. We showed that 60 μM CGA in alginate exhibited notable effects on stimulating chondrogenesis *in vitro*. Secondly, it was shown that the application of these complexes accelerated the recovery of injury-induced dysfunction by gait analysis when followed for 21 days. Histochemical analysis demonstrated that there was less abnormal vasculature formation, more chondrocyte proliferation and cartilage matrix synthesis in the presence of the complexes containing CGA. We discovered CGA treated transplantation up-regulated the expressions of Sox9 and Col2a1 which were responsible for the stimulation of chondrogenesis. Furthermore, the application of these complexes could suppress the abnormal angiogenesis and fibrosis at the injury site. Lastly, the elevated levels of inflammatory cytokines IL-1β, TNF-α, p-p65, and MMPs expression were decreased in the presence of CGA. This may be caused through adjusting cellular redox homeostasis associated with Nrf2. This study suggests that combining chondrocytes and CGA on an alginate scaffold can improve the recovery of damaged articular cartilage.

## Introduction

Articular cartilage is a white flake-like smooth tissue covering on the ends of bones at joints. Healthy cartilage in human joints allows the bones to glide smoothly over each other with minimal friction. Normal wear, tear, traumatic injury, or degenerative joint disease can lead to the damage of articular cartilage. Restoring articular cartilage can relieve patients’ pain, prevent the onset of osteoarthritis (OA) and can improve injured joint function.

Clinically, articular cartilage defects have plagued orthopedists, due to hyaline cartilige’s poor spontaneous healing potential. Various novel tissue-engineering techniques are evolving to help repair damaged cartilage through the stimulation of cartilage growth [1–3]. Generally, these strategies use the combination of stem cells or isolated chondrocytes with bio-materials as scaffolds to promote chondrogenic differentiation and cartilage growth.

The optimal choice is autologous cells because there is no risk of immunological rejection and infectious disease transmission [4, 5]. Additionally, much attention should be given to the viability and proliferative capacity of chondrocytes, as they are essential for the successful recovery of function of hyaline cartilage. It was previously shown that fetal or neonatal chondrocytes can be used in tissue grafts. This has been confirmed in animal models, in which articular cartilage was regenerated after neonatal chondrocyte transplantation [6].

The ideal bio-degradable scaffold should have a three-dimensional (3D) shape and appropriate mechanical strength. Moreover, the scaffold should ensure uniform cell concentration, promote chondrogenesis of the grafted cells, and avoid ectopic growth of cells from the articular cartilage defect. Given their properties, polylactic and polyglycolic acids quite often act as the chondrocytes carrier [7, 8]. Likewise, alginate has also been shown to be suitable for the cell-carriers in *in vivo* and *in vitro* experiments [9, 10]. This is partly due to alginate having a simulative effect on the chondrogenesis of transplanted chondrocytes [11]. Recent research highlighted that alginate-based hydrogels at the nanoscale by Layer-by-Layer assembly, are ideally served as scaffold in chondral regeneration in terms of their integrity, stability, and cytocompatibility [12].

The biochemical signals widely used are growth factors such as transforming growth factor β (TGF-β), insuluin like growth factor-1 (IGF-1) and fibroblast growth factor-2 (FGF-2) that are identified as functional stimuli to promote chondrogenesis in cultures [13, 14]. However, these growth factors are costly and systematically functionalized. Given these limitations, there is an increasing demand for more effective alternatives. Cartilage defects can develop from injury and also cartilage degradation, which is the major pathologic alteration in OA. Accumulating evidence has indicated that plant-derived small molecular compounds can cause distinctive effects on inhibiting inflammation caused by injury, and suppressing cartilage degradation. As one of the most plentiful polyphenols in diet, chlorogenic acid (CGA) is a cinnamic acid derivative and can be separated from natural plants including green coffee and beans. The main CGA compounds present in green coffee are readily absorbed and metabolized in the body. The biological effects of CGA are greatly associated with their antioxidant and anti-inflammatory properties [15, 16]. Of note, CGA has also been shown to reduce matrix metalloproteinases (MMPs) expression in chondrocytes [17]. Building on these properties, CGA is an ideal agent to suppress degenerative inflammatory processes.

In this study, we employed the combination of CGA in an alginate-scaffold containing chondrocytes to help restore cartilage function using an osteochondral defect model in the chick. The *in vitro* and *in vivo* experimental results identified that this combination efficiently regenerated hyaline cartilage and promoted the recovery of injury-induced dysfunction. These results suggest that CGA may serve as a potential agent to promote cartilage damage repair, through regulating chondrocyte proliferation, differentiation, extracellular matrix (ECM) synthesis, and suppressing inflammation by adjusting cellular redox homeostasis.

## Methods

### Isolation and culture of chick articular chondrocytes

Fertilized Leghorn eggs were obtained from the Avian Farm of South China Agriculture University (Guangzhou, China) and incubated in a humidified incubator (Yiheng Instruments, China) at 38°C until the desired embryonic stage. Primary cultured chondrocytes were isolated from the articular cartilage of 15-day chick embryos according to a previous published protocol with modification [18]. Briefly, the articular cartilage was washed three times, cut into 1 mm^3^ cubes, digested with 0.25% trypsin at 37°C with shaking for 1.5 hours, followed by collagenase type I for 6 hours. Next, the isolated chondrocytes were plated on a petri dish with DMEM (GIBCO, Invitrogen) supplemented with 1% (v/v) penicillin/streptomycin sulfate (GIBCO, Invitrogen), 1% (v/v) L-glutamine (GIBCO, Invitrogen) and 10% (v/v) fetal bovine serum (FBS, GIBCO, Invitrogen) at 37°C in a 5% CO_2_ humidified incubator. The 3^rd^ passage chondrocytes were reserved for implementation during the experiments.

### Cell counting kit-8 (CCK8) assay

The viability of the chondrocyte culture was assessed using a CCK8 assay (Dojindo Molecular Technologies, Japan). The chondrocytes were re-suspended with a density of 2×10^3^ cells/mL in 96-well plates, and treated with CGA at different concentrations (0, 15, 30, 60, 120, and 240 μM). After 24, 48, and 72 hours, 10 μL of CCK8 (5 g/L) was added, followed by 3 hours of incubation at 37°C. The absorbance values were then measured at 450 nm using a Bio-Rad Model 450 Microplate Reader (Bio-Rad, USA). Cell viability was indirectly established using the ratio of the absorbance value of CGA-treated cells relative to the control. The final results were representative of three independent experiments.

### Encapsulation of chondrocytes in alginate containing CGA

The culture of the 3D chondrocyte-alginate complex was based on a method modified which was previously described [18]. The prepared chondrocytes were re-suspended in 2% (w/v) alginate acid sodium (Sigma, USA) solution to make a suspension at 1×10^8^ cells/mL concentration. Meanwhile, CGA was added to achieve a final concentration of 60 μM. Then the 20 μL alginate/cell mixture drops were added into a sterile 102 mM Calcium Chloride (CaCl_2_, Sigma, USA) solution in a 6-well culture plate. CaCl_2_ was washed off by PBS after the 3D alginate-chondrocytes complexes had formed. Next the alginate-chondrocytes beads were added into the culture medium with 60 μM CGA, for a further 21-day culture in the incubator at 37°C. The medium was changed every 3 days. These chondrocyte-alginate complexes were further used for evaluation of mRNA expression, as well as histological staining.

### Chick injury model

Fertilized Leghorn eggs were incubated until the chicks hatched. The 14-day old chicks were used in the subsequent experiments. The chick injury model was established according to a method described previously [18]. All animal experiments were in accordance with the Guidelines on the Care and Use of Animals for Scientific Purposes (2004, Singapore). The protocols for the animal studies were also reviewed and approved by the Experimental Animal Ethical Committee of Jinan University (Permit number: 20160611001). Briefly, 14-day leghorn chicks were sterilely anesthetized through intravenous injection of 5 mg/kg xylazine (Sigma, USA). Under the stereoscope, the lateral condyle of right femur was exposed, and an osteochondral defect with 1 mm diameter and 2 mm depth was made using a 21 G needle. Then the osteochondral defects were implanted with the 3D chondrocytes-alginate complexes incorporated with or without 60 μM CGA. Six chicks were used for each group. After surgery, the animals were administered analgesics (Buprenorphine HCl, 0.02 mg/Kg/day, Sigma, USA) for three consecutive days. Gait analysis was performed on days 7, 14, 21 after the operation. The foot of the control side (left) was dyed with blue ink, while the injured right side was dyed in red before allowing the chicks to walk on white paper. Then assessments about stride length, stance length and sway length were carried out. Subsequently, harvesting of the surgical site was performed on post-surgery day 21. The animals were sacrificed under anaesthesia with 2% (v/v) isoflurane. Three samples were obtained from the articular surface of the lateral femoral condyle and fixed in 4% paraformaldehyde (PFA) for 48 hours at 4°C, then decalcified for 15 days before histological processing and staining. For the other 3 animals, the lateral 1/3 of the distal femur where the surgical operation had been previously performed, was removed and used to isolate RNA and protein for further analysis.

### Histochemical and immunofluorescent staining

The complexes of chondrocytes encapsulated in alginate were fixed in 4% PFA for 2 hours at 4°C after being cultured for 21 days, and dehydrated in a sucrose solution, and embedded in OCT. This was serially sectioned at 4 μm on a cryostat microtome (Leica CM 1850, Germany). Hematoxylin and Eosin (H&E) as well as PicroSirius red (Abcam, USA) staining were performed based on the standard protocol. The presence of sulfated proteoglycans was detected by staining with 0.1% Safranin O (Sigma, USA). The blood vessel density and the cell numbers at the defect site were measured and quantified by Image Pro-Plus (IPP 5.0, Media Cybernetics). The integral optical density (IOD) of Safranin O and the PicroSirius red staining area were also measured and compared.

Immunofluorescent staining on the primary cultured chondrocytes and chondrocyte-alginate complexes were performed using phospho-Histone3 (pHIS3; 1:200, Santa Cruz, USA), Sox9 (1:200, Sigma, USA), and Nrf2 (1:200, DSHB, USA) antibodies. Briefly, the fixed chondrocytes and chondrocyte-alginate complexes were incubated with the primary antibodies at 4°C overnight. After rinsing in PBST (0.1% Tween-20), the complexes were treated with the corresponding Alexa Fluor^®^ 555 or 488 labeled secondary antibody (1:1000, Invitrogen, USA) for 2 hours at room temperature, All the complexes were then later counterstained with DAPI (1:1000, Invitrogen, USA) at room temperature for 20 mins. For F-actin detection, cultured chondrocytes were stained using phalloidin-Alexa Fluor 555 (1:500, Invitrogen, USA) at room temperature for 2 hours.

The regions of interest were photographed using an Olympus IX51epi-fluorescent microscope and analyzed using CW4000 FISH Olympus software.

### RNA isolation and quantitative PCR (q-PCR)

Total RNA was extracted from the lateral 1/3 of the distal right femur using a Trizol kit (Invitrogen, USA) according to the manufacturer’s instructions. First-strand cDNA was synthesized to a final volume of 25 μL using SuperScript III First—Strand Synthesis SuperMix (Invitrogen, USA). Following reverse transcription, PCR amplification of the cDNA was performed using chick specific primers as previously described [19, 20]. Primer sequences are provided in S1 Fig. PCR reactions were performed in a Bio-Rad S1000TM Thermal cycler (Bio-Rad, USA) and ABI 7000 thermal cyclers, respectively. The housekeeping gene GAPDH was run in parallel to confirm that equal amounts of RNA used in each reaction. The expression of the genes was normalized to GAPDH, and the expression level was compared by ΔΔCt. The q-PCR result was representative of three independent experiments.

### Western blotting

Western blotting was performed in accordance with standard procedure using antibodies which specifically recognized MMP-13 (1:1000, Abcam, USA) and phospho-p65 (Ser536, 1:1000, Cell Signaling, USA). The protein was isolated using a radio-immuno-precipitation assay (RIPA, Sigma, USA) buffer supplemented with protease and phosphatase inhibitors. Protein concentrations were quantified with the BCA assay. The loading control was a β-actin antibody (1:3000, Proteintech, USA). Quantity One (BIO-RAD, USA) was used to capture the chemiluminescent signals and analyze the data. All samples were performed in triplicate.

### Measurement of reactive oxygen species (ROS)

To assess the extent of oxidative stress, the chondrocytes were homogenated and then the levels of intercellular ROS were measured using a non-fluorescent dye, 2’,7’-dichlorodihydrofluorescein diacetate (DCFH-DA, Sigma, USA), which is oxidized by ROS into a fluorescent dye, 2’,7’-dichloroflurescin (DCFH). Furthermore, lipopolysaccharide (LPS) and vitamin C were employed as a ROS inducer and scavenger, respectively, to verify the mechanism of CGA on oxidative stress. Briefly, the primary cultured chondrocytes were treated with LPS (10 μg/mL, Sigma, USA), and LPS + CGA for 24 hours respectively, then incubated in presence of 10 μM DCFH-DA for 20 minutes. The fluorescence was measured using BD FACSAria (BD Bioscience, USA). The total RNA was extracted for later analysis. All experiments were carried out in triplicate.

### Data analysis

The data produced are presented as mean ± standard error (SE). ANOVA tests were performed using Graphpad Prism 5 (Graphpad Software, USA). Samples were considered to be significantly different from control when *p*<0.05, and extremely significantly different when *p*<0.01.

## Results

### Determining the optimal concentration of CGA for application in the alginate scaffold

**Fig 1:**
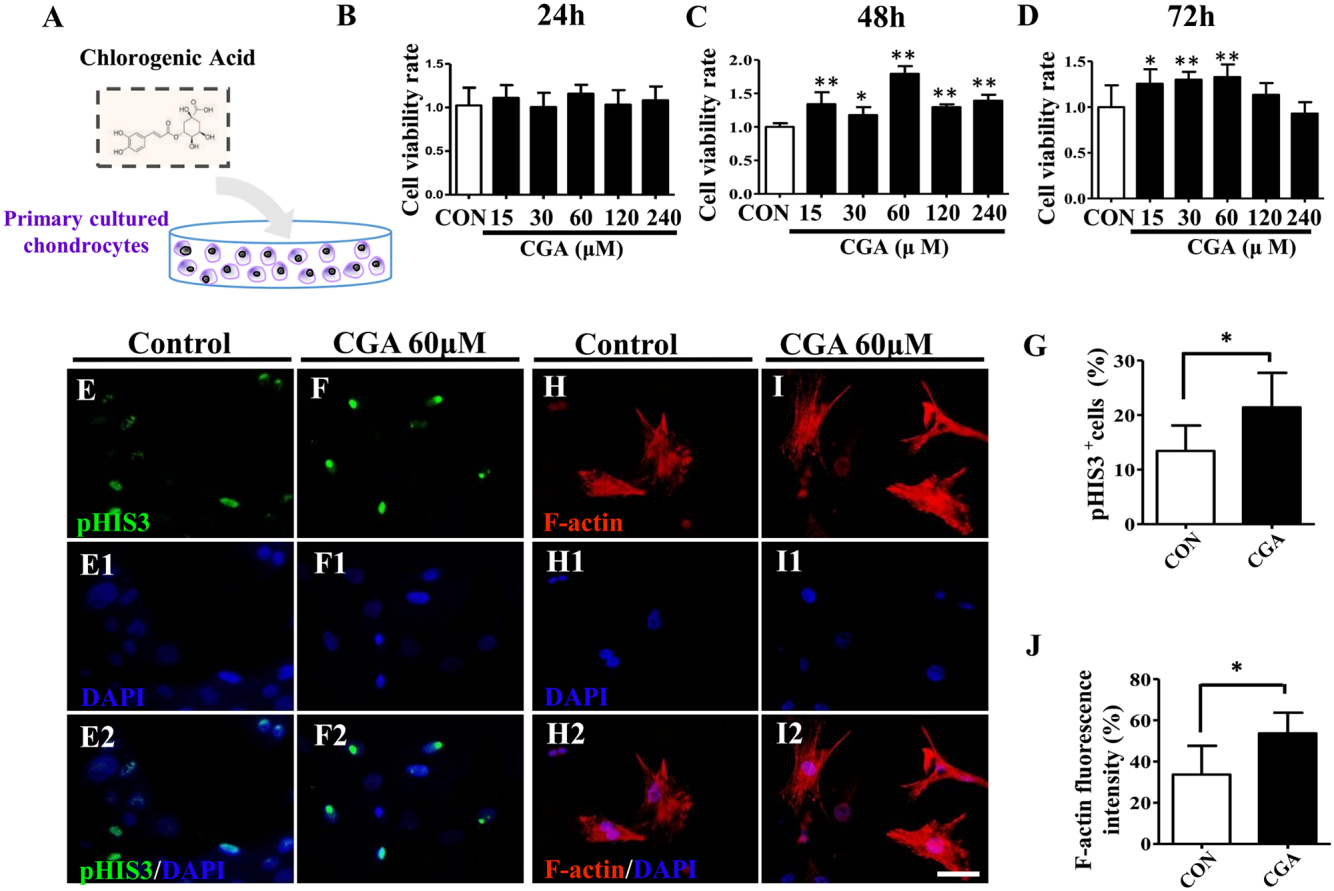
*In vitro* assessment of the optimal CGA concentration using a primary culture of chick chondrocytes. (**A**) Sketches illustrating the primary culture of chondrocytes isolated from embryonic 15-day chick articular cartilage in the presence of CGA *in vitro* (see Materials and methods for more details). (**B-D)** CCK8 assays were carried out to detect the cell viability following *in vitro* primary culture of chondrocytes at 24 (B), 48 (C) and 72 (D) hours in the absence and presence of different concentrations of CGA. (**E-F, E1-F1, E2-F2)** pHIS3 immunofluorescent staining on 48h-cultured chondrocytes in the absence (E) or presence (F) of 60μM CGA. E1-F1 are with DAPI staining while E2-F2 are the merged images of pHIS3 immunofluorescent staining and DAPI. (**H-I, H1-I1, H2-I2)** F-actin fluorescent staining on 48h cultured chondrocytes in the absence (H) or presence (I) of 60μM CGA. H1-I1 are with DAPI staining while H2-I2 are the merged images of F-actin staining and DAPI. (**G)** Bar chart showing the ratio of pHIS3^+^ cells between controls and CGA-treated groups. (**J)** Bar chart showing the ratio of F-actin fluorescent intensity between controls and CGA-treated groups. Scale bars = 20μm in E-I, E1-I1, E2-I2.

The primary cultured chondrocytes were incubated in both the absence and presence of various concentrations of CGA (15, 30, 60, 120 and 240 μM) (Fig 1A–1D). The CCK8 assay was employed to determine the cell viability of chondrocytes. This determined that 60 μM CGA could effectively enhance the viability of chondrocytes at 24-, 48- and 72-hour (Fig 1B–1D). There was an especially significant increase in viability at the 48- and 72-hour time points (Fig 1C and 1D). Therefore, 60 μM CGA was used in the subsequent experiments. pHIS3 immunofluorescent staining demonstrated that there were increased cell numbers of chondrocytes incubated with 60 μM CGA when compared to controls (Fig 1E–1F2 and 1G). F-actin fluorescent staining on the primary cultured chondrocytes showed that 60 μM CGA noticeably stimulated cell extension of the chondrocytes (Fig 1H–1I2 and 1J).

### CGA promotes chondrogenesis *in vitro*

**Fig 2:**
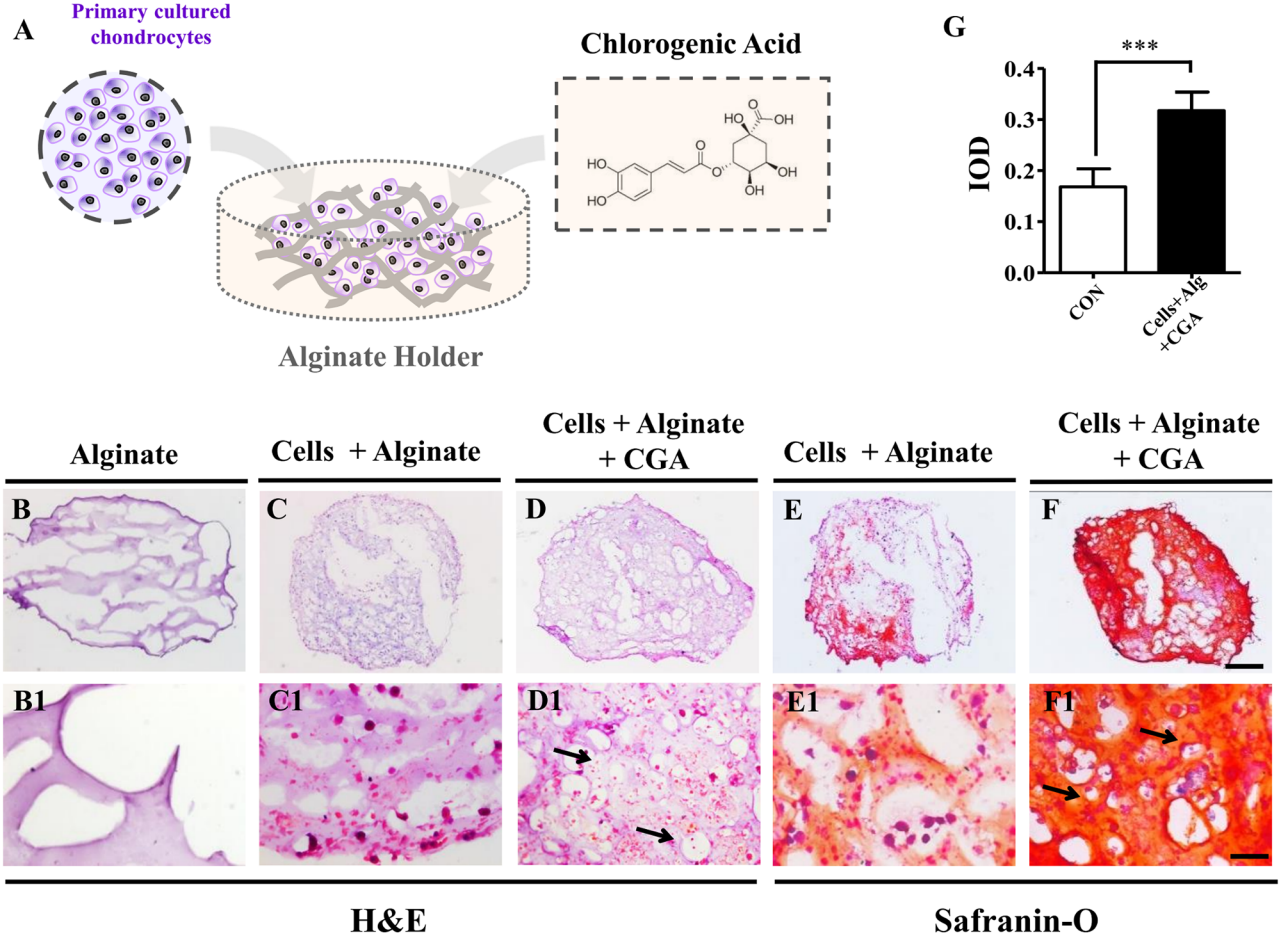
The assessment of chondrogenesis following the application of CGA and alginate. (**A**) Sketches illustrating chondrocytes isolated from the embryonic 15-day chick articular cartilage in alginate with 60μM CGA. (**B-D)** H&E staining on the 21-day culture with alginate only (B), chondrocytes + alginate (C) or chondrocytes + alginate + CGA (D). (**B1-D1)** High magnification images from B-D respectively. **(E-F)** Safranin-O staining on 21-day cultured chondrocytes + alginate (E) or chondrocytes + alginate + CGA (F). (**E1-F1)** High magnification images from E-F respectively. (**G)** Bar chart showing the comparison of IOD from Safranin-O staining between controls and CGA groups. Scale bars = 500μm in B-F and 100μm in B1-F1.

3D compounds formed quickly after the chondrocytes-alginate suspension was crosslinked with the CaCl_2_ solution (Fig 2A). H&E staining after 21-days of incubation, showed that there were more cells and a denser extracellular matrix in the 60 μM CGA treated group (Fig 2D) when compared with the cells + alginate as a positive control (Fig 2C), while alginate without cells was used as a negative control (Fig 2B). This was more apparent with high magnification images (Fig 2B1–2D1). Safranin O is a cationic dye that combines with acidic proteoglycan present in cartilaginous tissue, therefore, Safranin O staining is used as an indicator of chondrogenesis, with its intensity being directly proportional to the proteoglycan content in cartilage. This staining demonstrated deeper red and orange in the CGA treated group (Fig 2F) than in the cells + alginate group (Fig 2E). Again, this difference was more distinctly shown by high magnification (Fig 2E1–2F1). Moreover, IOD data further demonstrated that the *in vitro* chondrogenesis was promoted by CGA in the alginate complexes (Fig 2G).

### Transplantation of chondrocytes treated with CGA improves the functional recovery of the injured chick knee joint

**Fig 3:**
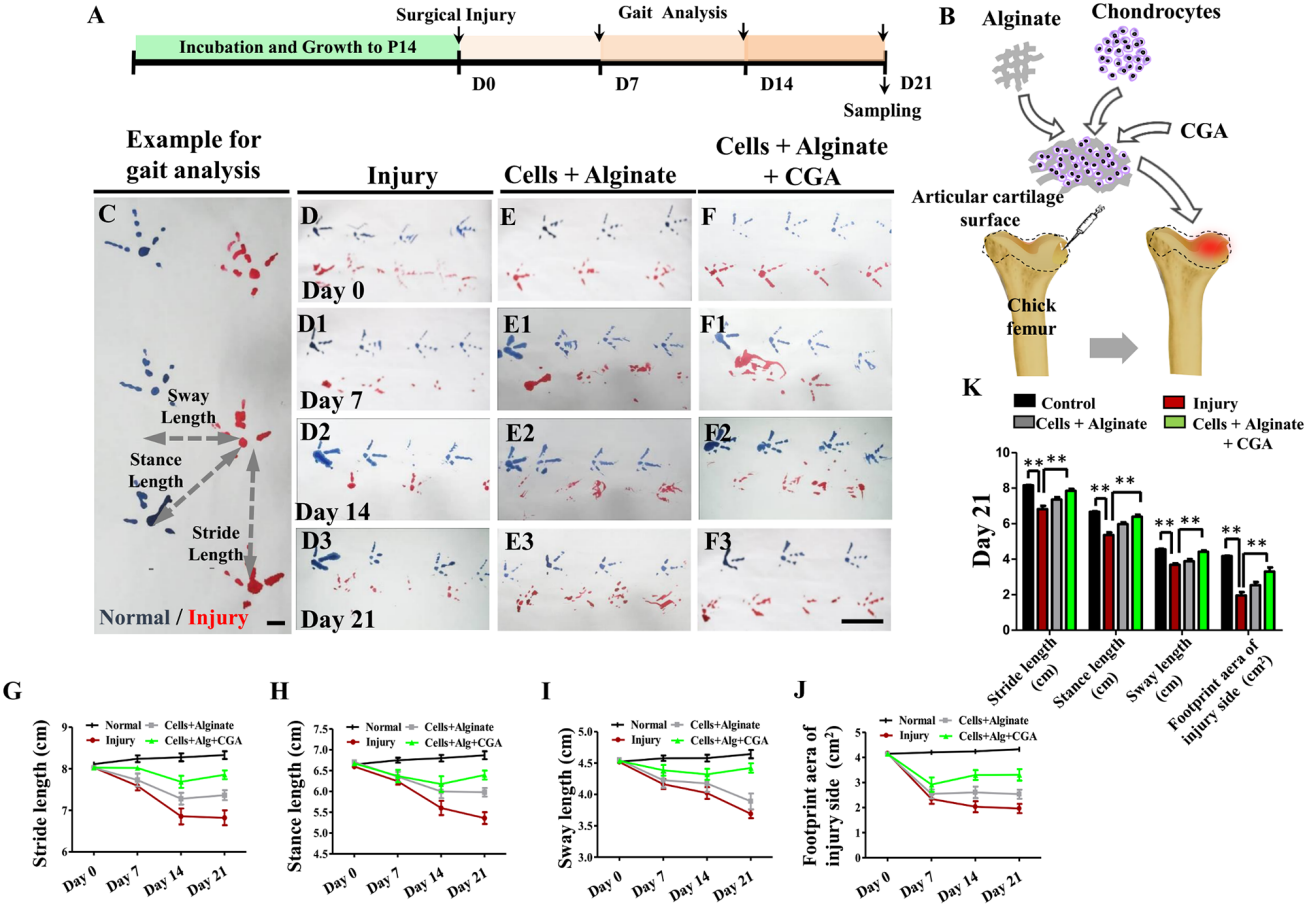
The assessment of articular function following the application of different complex combinations on injured chick knees. (**A-B**) Sketches illustrating the timing of knee joint injury on the 14-day post-hatched chick model and gait analysis following treatment (A), sketch demonstrating the injury model and the subsequent treatment (B). (**C)** An example of gait analysis, in which blue foot prints are normal and red ones are from the injured side. (**D-F, D1-F1, D2-F2, D3-F3)** Representative chick gaits on postoperative day 0 (D-F), 7 (D1-F1), 14 (D2-F2), 21 (D3-F3) of control, alginate, and CGA treated groups. (**G-J)** Graphs showing the chick gait analysis highlighting stride length (G), stance length (H), sway length (I) and footprint area of the injured side (J) on days 0, 7, 14, and 21 following treatment. (**K)** Bar chart of the chick gait analysis among the control, alginate, and CGA treated groups on day 21 after the treatment. Scale bars = 1cm in C and 5cm in D-F, D1-F1, D2-F2, D3-F3.

As illustrated in Fig 3A and 3B, injured chick knee joints (Fig 3B) were employed as a cartilage defect model to evaluate the application of chondrocytes treated with CGA on functional improvement and the recovery of damaged articular cartilage. Gait analysis including stride length, stance length, sway length, and footprint areas (Fig 3C) were chosen as functional indices to estimate the therapeutic effects. Analysis was done on days 0, 7, 14 and 21 following the transplantation of the chondrocyte-alginate-CGA scaffold. There was minimal effect on stride length and stance length on day 0 (Fig 3D–3F), but there was eventually a difference for both on days 7 (Fig 3D1–3F1) and 14 (Fig 3D2–3F2). The change was most obvious on day 21 (Fig 3D3–3F3). The combination of chondrocytes, CGA and alginate significantly improved the injury-induced gait disorder (Fig 3G–3K). Also noticeable was the improvement of stride length and sway length which occurred earlier (day 7) (Fig 3G–3J). Taken together, the application of chondrocytes, alginate and CGA improved the functional repair of articular cartilage in the injured chick model.

### Confirming the effectiveness of the application of chondrocytes and CGA in alginate using histological analysis

**Fig 4:**
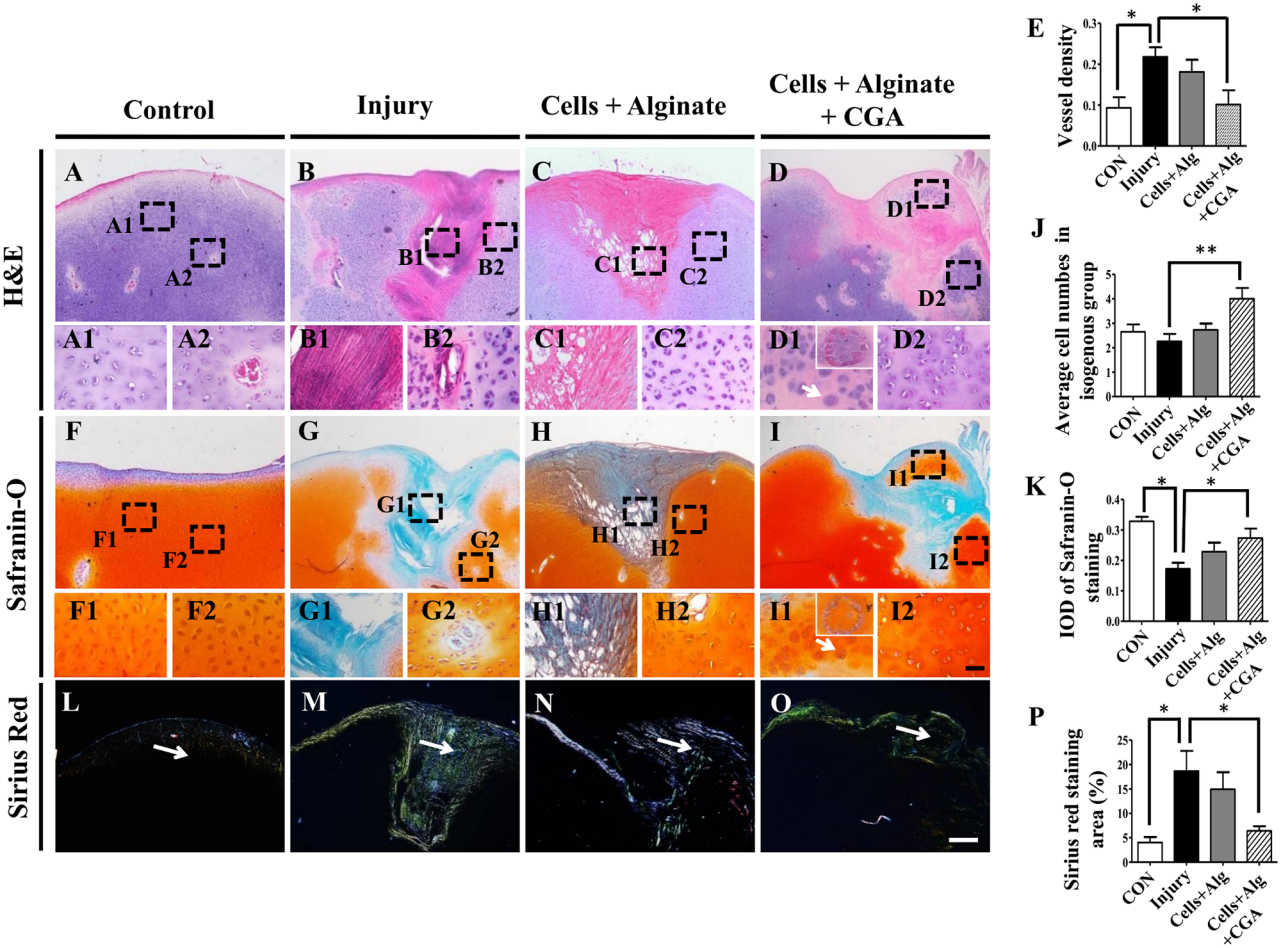
The assessment of articular histology following different treatments on injured chick knees. (**A-B**) The representative H&E staining on longitudinal sections of the injured chick knees from a normal control (A), a model control (B), after the application of chondrocytes + alginate (C), or the application of chondrocytes + alginate + CGA (D). (**A1-A2, B1-B2, C1-C2, D1-D2)** High magnification images from the sites indicated by dotted squares in A-D respectively. (**F-I)** Representative Safranin-O staining on longitudinal sections of the injured chick knees from the normal control (F), the model control (G), and after the application of chondrocytes + alginate (H), or the application of chondrocytes + alginate + CGA (I). (**F1-F2, G1-G2, H1-H2, I1-I2)** High magnification images from the sites indicated by dotted squares in F-I respectively. (**L-O)** Representative Sirius red staining on the longitudinal sections of injured chick knees from the normal control (L), the model control (M), and after the application of chondrocytes + alginate (N), or the application of chondrocytes + alginate + CGA (O). (**E, J, K, P)** Bar charts showing the comparison of blood vessel density (E), average cell numbers in isogenous groups of chondrocytes (J), IOD of the articular cartilage (K), Sirius red staining area percentage (P) among the different treatment groups. Scale bars = 300μm in A- D, F-I, L-O, 50μm in A1-D1, F1-I1, A2-D2, F2-I2.

H&E, Safranin-O or PicroSirius red staining were implemented on the sections of the injured chick knee joints on day 21 after different treatments. H&E staining demonstrated that the wounds on the articular cartilage were healing in all groups, (Fig 4A–4D). However, when comparing the injury group controls and the CGA group (Fig 4B1, 4B2, 4C1 and 4C2), there were more cellular components at the healing sites of the CGA group (Fig 4D1 and 4D2). Additionally, injury-induced angiogenesis was inhibited by CGA (Fig 4E), and more isogenous groups such as cellular aggregates were observed in the CGA group (as indicated by arrows in Fig 4D1 and 4J). We also demonstrated that Safranin-O positive cartilage matrix was enhanced by CGA as shown with the dotted squares (Fig 4F, 4I, 4F1, 4I1, 4F2 and 4I2). The average IOD of Safranin-O staining at the injury site in the CGA group was higher than those of the injury alone group and cell + alginate without CGA group (Fig 4K). Furthermore, PicroSirius red staining showed that the fibrosis induced by injury (Fig 4M and 4N) was dramatically inhibited by CGA (Fig 4O and 4P). These data suggest that the application of CGA to chondrocytes in an alginate scaffold could stimulate *in vivo* chondrogenesis as well as inhibit injury-induced fibrosis.

### CGA improved chondrogenesis and inhibited inflammation in the chick knee injury model

**Fig 5:**
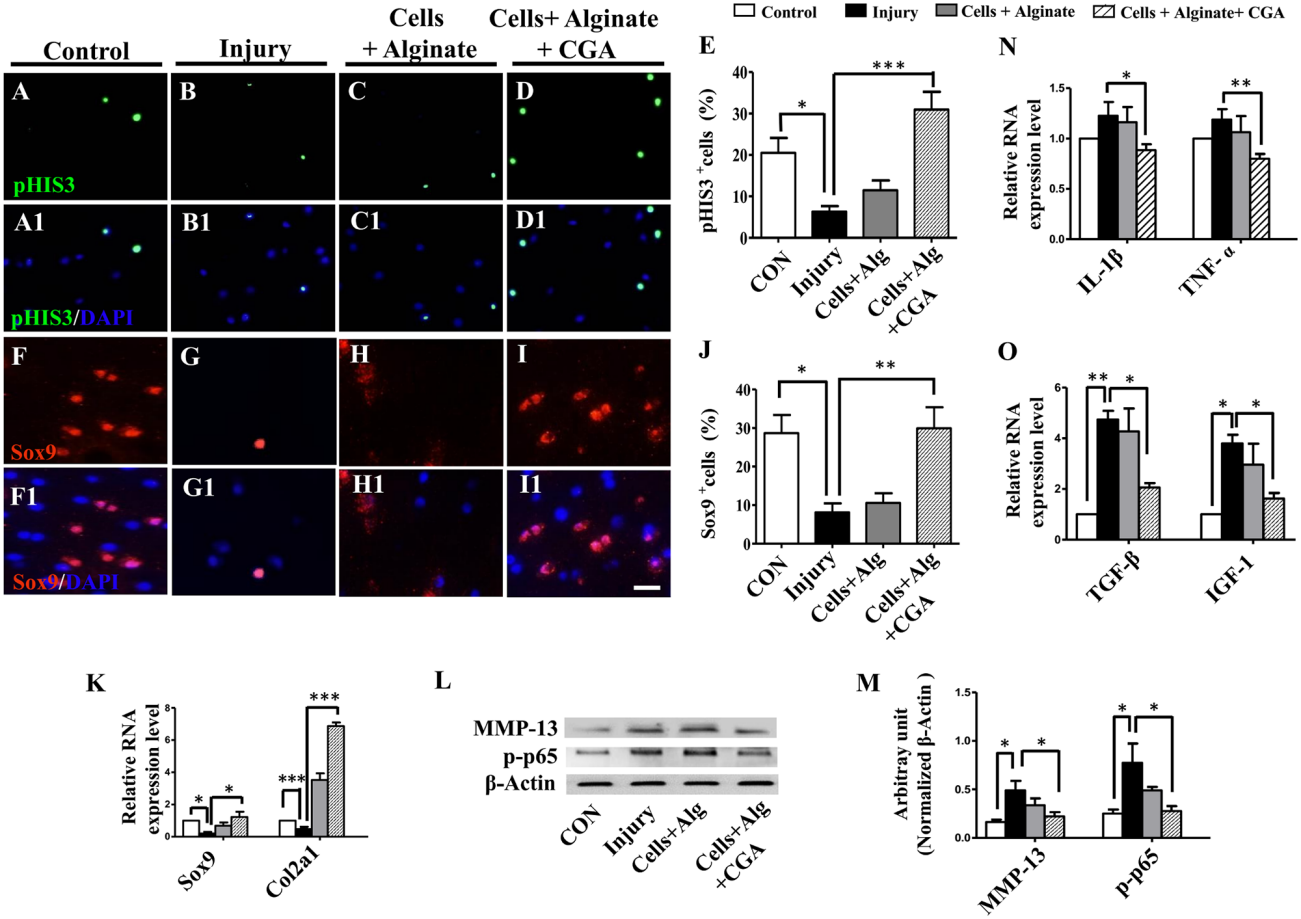
Determining the effect of different combinations on chondrocyte proliferation on injured chick knees. (**A-D**) Representative pHIS3 immunofluorescent staining on the sections of the repairing articular cartilage from the normal control (A), the model control (B), and after the application of chondrocytes + alginate (C), or application of chondrocytes + alginate + CGA (D). (**A1-D1)** Merged pHIS3 and DAPI staining of A-D respectively. (**F-I)** Representative Sox9 immunofluorescent staining on sections of the healing articular cartilage from the normal control (F), the model control (G), and after the application of chondrocytes + alginate (H), or the application of chondrocytes + alginate + CGA (I). (**F1-I1)** Merged Sox9 and DAPI staining of F-I respectively. (**E, J**) Bar charts showing the ratio of pHIS3^+^ cells (E) and Sox9^+^ cells (J) of total DAPI^+^ cells among different treatment groups. (**K)** q-PCR results of the expressions of Sox9 and Col2a at the mRNA level among different treatment groups. (**L-M)** Western blot data of the expressions of MMP-13 and p-p65 at the protein level among the different treatment groups. (**N-O)** q-PCR results showing the expressions of IL-1β, TNF-α, TGF-β and IGF-1 at the mRNA level among the different treatment groups. Scale bars = 10μm in A-D, F-I, A1-D1 and F1-I1.

To investigate the underlying mechanism for the improved functional recovery with CGA, pHIS3 and Sox9 immunofluorescent staining was performed on sections at the cartilage injury site. This showed that injury reduced the pHIS3^+^ cell numbers compared with controls (Fig 5A, 5B, 5A1, 5B1 and 5E), while transplantation of chondrocytes with CGA increases the pHIS3^+^ cell numbers(Fig 5D, 5D1 and 5E). This demonstrated that the proliferation of chondrocytes was activated by the alginate-CGA complexes. Sox9 plays an important role on the maintenance of chondrocyte proliferation [21]. Given this fact, we investigated the expression of Sox9 in our injury model. It was shown that injury inhibited cell proliferation (Fig 5F, 5G, 5F1, 5G1 and 5J) and the number of Sox9^+^ cells were not changed by the simple transplantation of chondrocytes (Fig 5H, 5H1 and 5J). However, the transplantation of chondrocytes treated with CGA almost returned the Sox9^+^ cell numbers to a pre-injury level (Fig 5I, 5I1 and 5J). Furthermore, q-PCR data demonstrated a similar pattern of Sox9 and Col2a1gene expression (Fig 5K).

Additionally, it is accepted that excessive production of MMPs can contribute to cartilage breakdown as well [22, 23]. Western blot data showed that MMP-13 expression was increased by injury, but returned to near control levels after the application of chondrocytes with CGA in alginate (Fig 5L and 5M, S2 Fig). In total, this implied that the *in vivo* application of a complex of chondrocytes, CGA, and alginate could enhance chondrocyte proliferation and decrease ECM degradation.

To further investigate the improved recovery seen with CGA treated chondrocyte transplantation, we examined the gene expressions of inflammation cytokines IL-1β (interleukin-1β), TNF-α (tumor necrosis factor α) and TGF-β. This showed that injury increased the expression of IL-1β, TNF-ɑ and TGF-β, and their expression significantly decreased after the addition of chondrocytes treated with CGA (Fig 5N and 5O). The expression of p-p65 (phospho-NF-κB p65) was increased by injury and also decreased with treatment (Fig 5L and 5M, S2 Fig).

### Evaluating the mechanism underlying the inhibition of fibrosis by CGA treated chondrocytes

**Fig 6:**
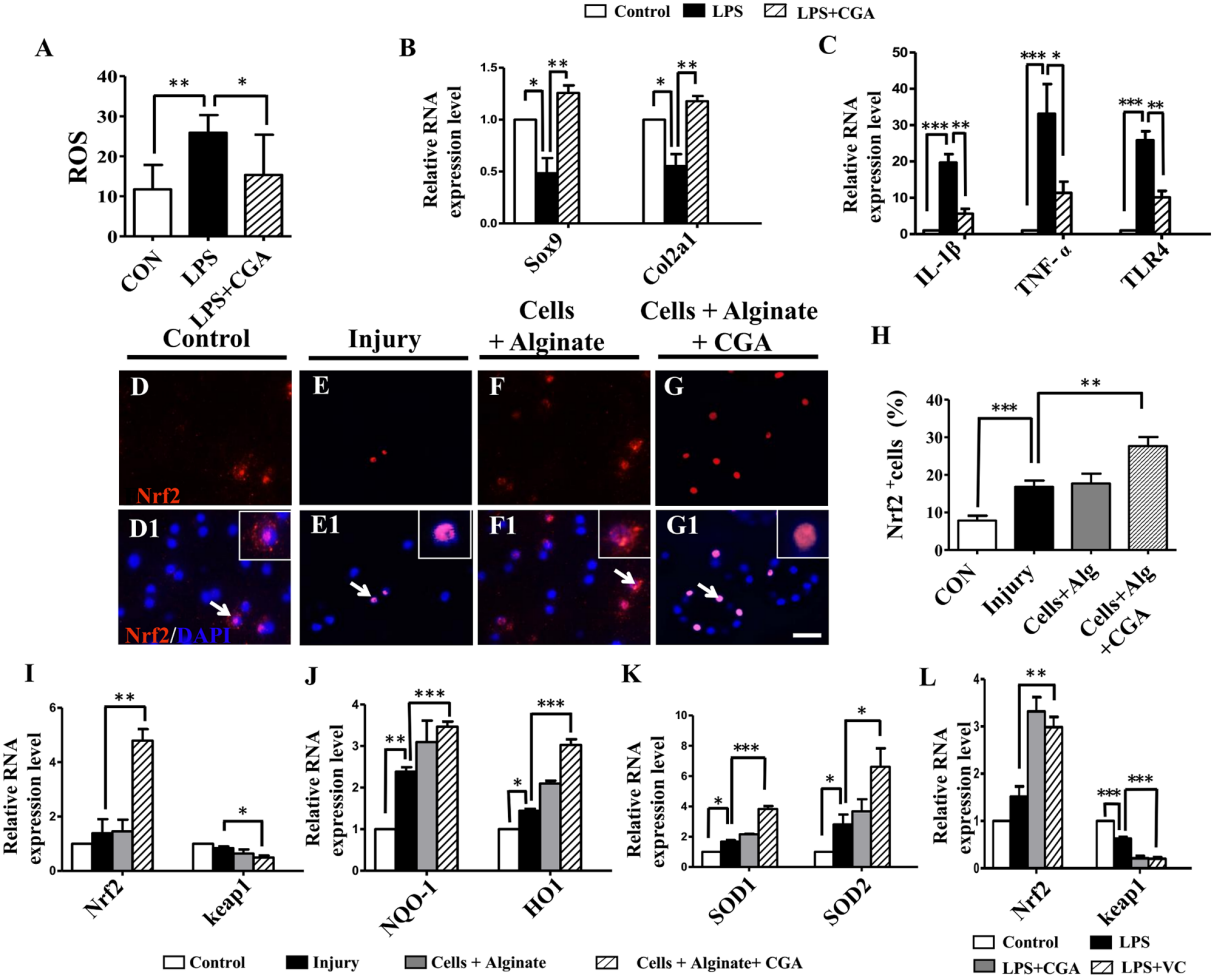
Determining the effect of the different matrix combinations on the inflammation and oxidative stress on injured chick knees. (**A**) Bar chart showing the comparison of the ROS production in chondrocyte cultures among the control, the LPS-treated and the LPS+CGA-treated groups. (**B-C)** q-PCR of the expressions of Sox9, Col2a and IL-1β, TNF-α, TLR-4 at the mRNA level in chondrocyte cultures among the control, the LPS-treated and the LPS+CGA-treated groups. **(D-G)** Representative Nrf2 immunofluorescent staining on the transverse sections of healing articular cartilage from the control (D), the injury only (E), and after the application of chondrocytes + alginate (F), or the application of chondrocytes + CGA + alginate (G). (**D1-G1)** DAPI staining on the samples from D-G. (**H)** Bar chart showing the percentage of Nrf2^+^ cells of total DAPI^+^ cells among the different treatment groups. (**I-K)** q-PCR results of the expressions of Nrf2, keap1, NQO-1, HO-1, SOD1 and SOD2 at the mRNA level among the different treatment groups. (**L)** q-PCR of the expressions of Nrf2 and keap1 at the mRNA level in chondrocyte cultures among the control, the LPS-treated, the LPS+CGA-treated and the LPS + Vitamin C-treated groups. Scale bars = 10μm in D-G, D1-G1.

In an *in vitro* model of LPS-induced ROS production, we demonstrated a significant decrease in ROS when CGA was added into the culture medium (Fig 6A). q-PCR data showed that CGA reversed the LPS-induced reduction in expression of both Sox9 and Col2al (Fig 6B). The expression of the inflammatory cytokine TNF-ɑ, the fibrosis related TGF-β, and the immune activating TLR-4 were all decreased under the application of CGA when compared with the LPS-control (Fig 6C). The expression of nuclear factor erythroid 2-related factor 2 (Nrf2), a key regulator for an antioxidant response [24], was reduced in the injury model, and recovered with the CGA chondrocyte complex (Fig 6D, 6G, 6D1, 6G1 and 6H). We further checked oxidative stress related gene expression using q-PCR, which showed the expression of Nrf2, NQO-1, HO-1, SOD1and SOD2 were up-regulated. Only keap1 remained decreased (Fig 6I, 6J and 6K).

Together this suggests that the application of chondrocytes with CGA in alginate inhibited the inflammation-induced fibrosis, which is possibly accomplished through a re-balance of the system oxidation to anti-oxidation responses. Using the LPS-induced model for developing ROS, we demonstrated the anti-oxidant effects of CGA and confirmed that the gene expression was similar to those *in vivo*. This was further verified by the known anti-oxidant vitamin C, which exhibited the same effects on the expression of Nrf2 and keap1 caused by LPS exposure in a chondrocyte culture, as CGA demonstrated on the *in vivo* transplantation (Fig 6L).

## Discussion

CGA is an ester of caffeic acid and quinic acid, and is one of the most abundant polyphenols in the human diet, and is found in coffee, fruits and vegetables. There is increasing evidence in animal models showing the anti-oxidant and anti-inflammatory properties of CGA [25]. As such, it was employed as a biochemical signaling molecule in the alginate scaffold to help repair damaged articular cartilage. First we determined that 60 μM was the optimal concentration of CGA to maintain chondrocytes viability *in vitro* (Fig 1), which is higher than 20 μM of CGA concentration used in a previous report [17, 19].

To transplant the CGA-treated chondrocytes to articular cartilage *in vivo*, the cells must be seeded onto a carrier which facilitates the uniform distribution of chondrocytes and constrains cells to the injury site. To accomplish this, we used sodium alginate. Alginate is an anionic polysaccharide that is abundantly found in brown algae, and forms a viscous gum if exposed to water [11]. Alginate gel particles are frequently employed as hydrocolloid gel particles because they are biocompatible, nontoxic, biodegradable, cheap, and easily produced. Encapsulation of desired cells in an alginate gel particle is valuable due to the particle’s protective effect on DNA, nutrients, and protection from microbes. Obviously, the effectiveness of alginate gel particles relies on their physical properties in various conditions [26]. In this study, we found that alginate promoted the *in vitro* chondrogenesis to some extent (Fig 2). Next, we determined that the combination of chondrocytes and alginate in the presence of CGA promoted chondrogenesis *in vitro* more robustly (Fig 2). Ultimately, we determined that that application of these complexes can currently be accomplished with relative feasibility in an *in vivo* model. Following the application of the complexes in an *in vivo model*, we employed a gait test to serve as the functional index to measure the effect of treatment of articular cartilage damage. This gait analysis has been validated as a measure of musculoskeletal and central/motor neuron anomalies [27–29]. It has also been used to assess the effectiveness of surgical cartilage repair for chondral and bone defects [30, 31]. This allowed us to objectively measure the recovery effect of cartilage damage. We showed obvious improvement on the recovery of knee function with the application of the chondrocyte and CGA in alginate complex to the articular cartilage defect (Fig 3).

Histological analysis of the articular cartilage in our injury model, showed that the CGA complexes effectively reduced angiogenesis and stimulated chondrogenesis including chondrocyte proliferation and cartilage ECM synthesis (Fig 4). Ossification at the wound site inevitably happens if blood vessels invade into the injury site [32]. This highlights the importance of inhibiting injury-induced angiogenesis during wound healing as we have shown (Fig 4A–4K). It was also noteworthy that the injury-induced fibrosis has deleterious effects on cartilage function. We demonstrated that our complexes significantly inhibited fibrosis (Fig 4L–4P), which we postulated exerted a positive effect on the cartilaginous healing.

To further explore possible mechanisms of healing, we found that cell proliferation was promoted by CGA contained complexes at the wound site (Fig 5A–5E). Sox9 is a transcription activator required for chondrogenesis [33]. We demonstrated that Sox9 was up-regulated as well as a downstream gene Col2a1. We also demonstrated down regulated MMP-13 expression, a detrimental gene to healing, in the presence of CGA (Fig 5F–5M). This suggests that the CGA complexes employed in this study effectively stimulate *in vivo* chondrogenesis. This is generally consistent with previous report, showing that CGA treatment reduced MMP-1, MMP-3, and MMP-13 expression and increased TIMP-1 expression in cartilage [17].

Pathological wound repair often derives from excessive fibrosis at the site of injury. The ultimate result is that the healed tissue fails to regain normal function. Cytokines play a vital role in injuries and inflammatory responses which lead to fibrosis in the injured tissues [34]. Potent cytokines, IL-1β and TNF-α are capable of promoting MMPs and TIMP (tissue inhibitors of MMPs) synthesis [35, 36]. TGF-β is shown to increase cartilage degradation through anabolic factor IL-1 [37, 38]. The biological effects of TNF-α are frequently achieved through the coordination of the Nuclear Factor kappa B (NF-κB)/p65 signaling pathway [39]. In this study, we observed that the enhanced expressions of IL-1β, TNF-ɑ and p-p65 were suppressed by the complexes containing CGA (Fig 5L–5N). This implied that the injury-induced inflammation was effectively inhibited, which eventually was beneficial in the reduction of fibrosis during cartilage defect healing (Fig 5O).

Cellular redox homeostasis plays a vital role in the healing process by balancing the amount of intracellular ROS. Our results revealed that the CGA complexes could lower the LPS-induced increase of ROS. We verified this through the increased expression of Sox9/Col2a1 (Fig 6A–6C), as well as Nrf2 *in vivo* (Fig 6D–6H and 6I). Nrf2 serves a transcription factor that promotes the transcription of a variety of antioxidant genes as part of the cellular resistance to oxidative stress, exogenous toxic substances, and downstream molecules *in vivo* (Fig 6J–6K) [40].

**Fig 7:**
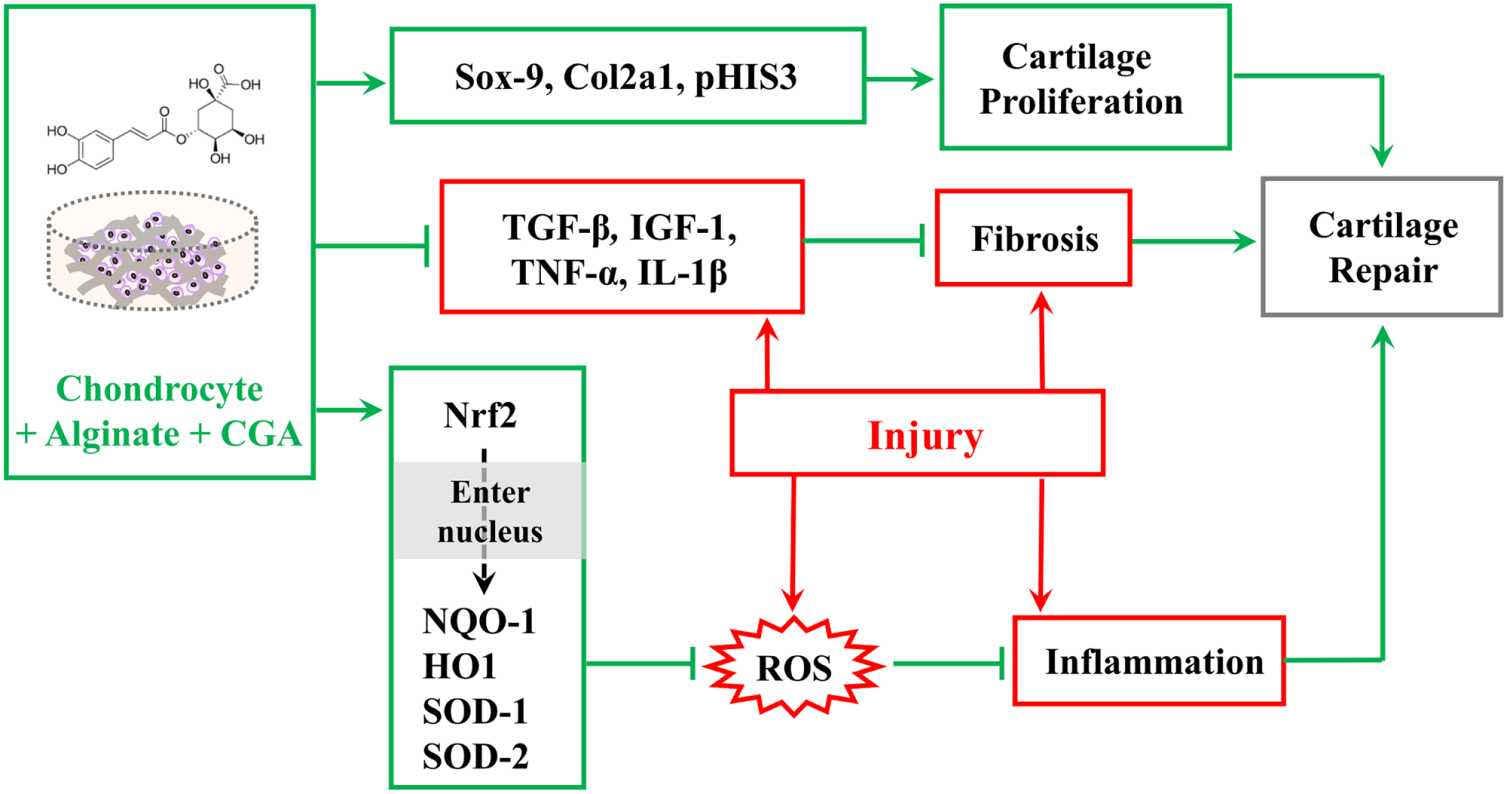
Model depicting how the complexes containing CGA improve the recovery of injured articular cartilage.

In summary, *in vivo* and *in vitro* experimental approaches reveal that the application of chondrocytes with CGA in alginate scaffold improved chondrogenesis and suppressed fibrosis. We speculated that the underlying mechanism for recovery might include two aspects. First, CGA stimulates chondrocyte proliferation and synthesizes cartilage matrix through the activation of Sox9 and Col2a1, as well as suppressing MMP-13 expression. Meanwhile, CGA in an alginate complex could also down-regulate the injury-induced increase of inflammatory cytokines such as IL-1β and TNF-ɑ, as well as p-p65 through maintaining cellular redox homeostasis. Ultimately, this was beneficial in suppressing fibrosis during wound healing, and conducive to improved articular cartilage function (Fig 7). Future studies should focus more on how to improve the chondrocyte scaffold’s effectiveness. Eventually, this may open up new doors for clinical treatment of articular cartilage damage.

## Supporting information

S1 Fig The sets of primers used for PCR in this study. (TIF)

S2 Fig The original western blots results of MMP-13 and p-p65 of Fig 5L. (TIF)
