# Supplementary figures and images for: Applying chlorogenic acid in an alginate scaffold of chondrocytes can improve the repair of damaged articular cartilage
Source: PLoS One. 2018 Apr 5;13(4):e0195326. doi: 10.1371/journal.pone.0195326 (PMC5886530; doi:10.1371/journal.pone.0195326)

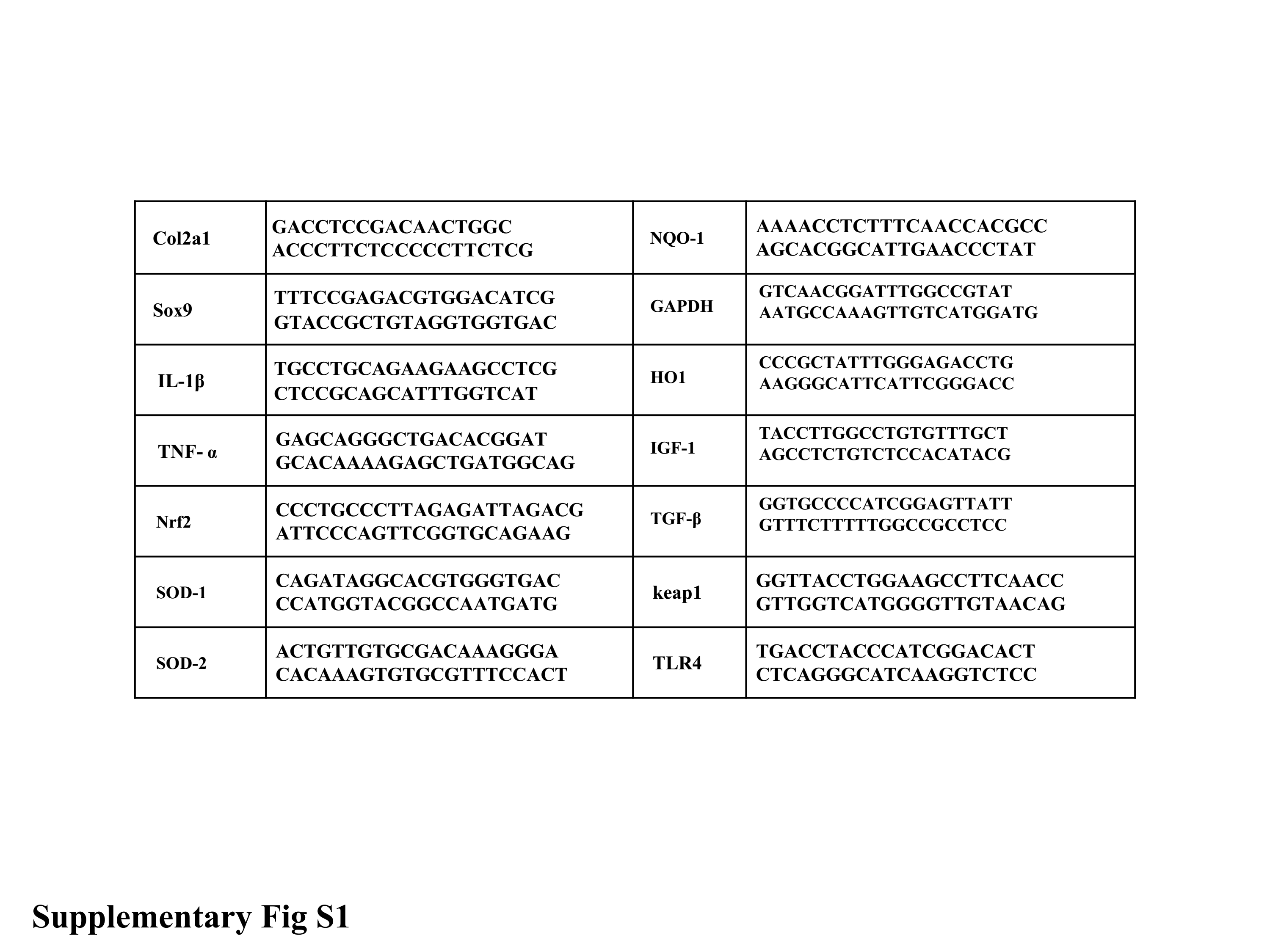

Supplement: S1 Fig — (TIF) [file pone.0195326.s001.tif]

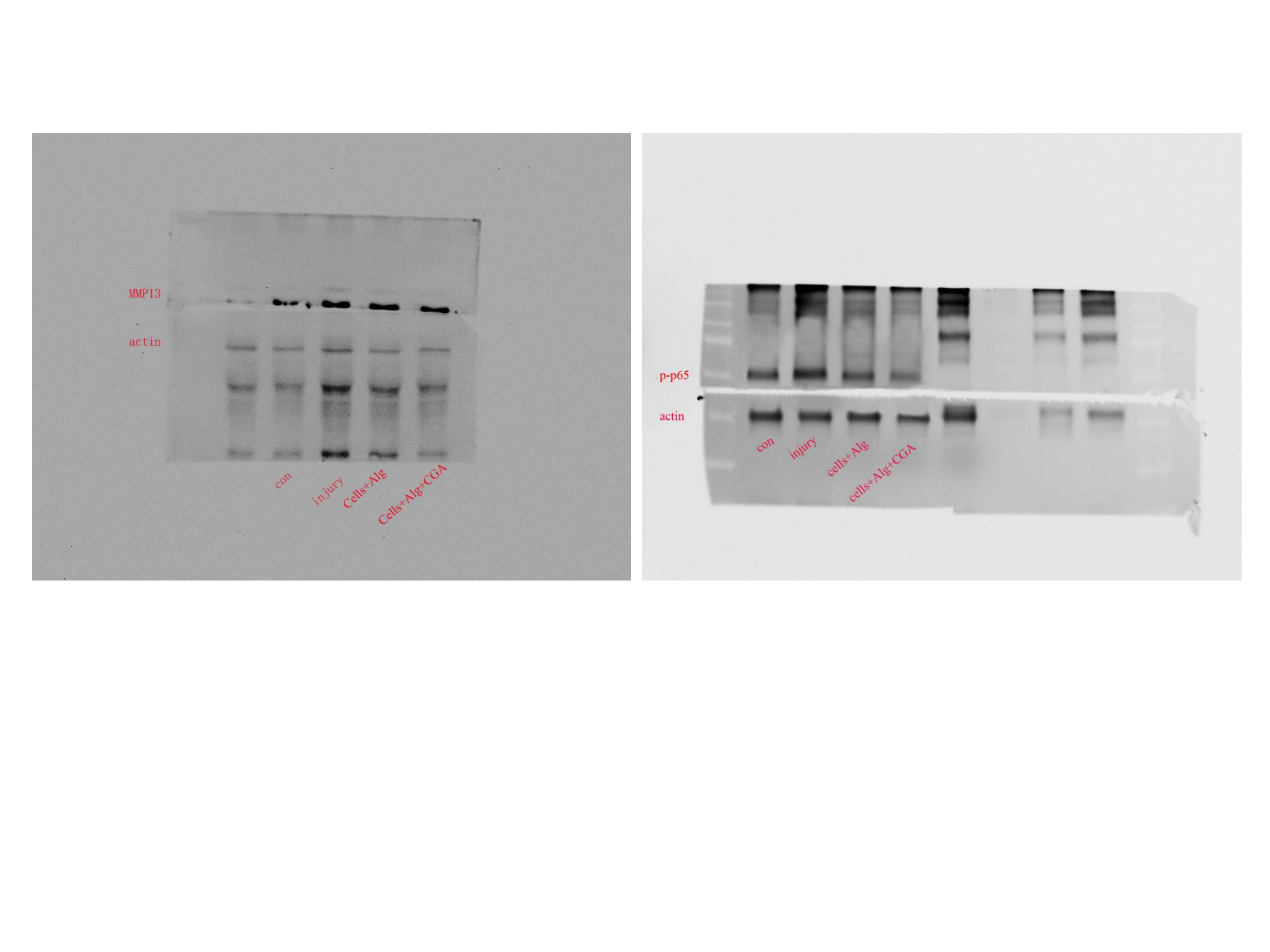

Supplement: S2 Fig — (TIF) [file pone.0195326.s002.tif]
